# Interactive effects of biochar, nitrogen fertilizer, and irrigation on SOC and crop yield in a rice-wheat rotation system

**DOI:** 10.3389/fpls.2026.1859708

**Published:** 2026-06-30

**Authors:** Danyan Chen, Yuanyuan Feng, Hailing Li, Ya Liu, Hongbo Ma, Xianju Xu, Xiangyu Kong, Heng Qi, Yuxuan Zhang, Jiaying Wang, Jingze Ma, Zhenyue Li, Jiameng Qian, Wei Zhou

**Affiliations:** 1 College of Horticulture, Jinling Institute of Technology, Nanjing, China; 2 Co-Innovation Center for Sustainable Forestry in Southern China, College of Forestry, Nanjing Forestry University, Nanjing, China; 3 School of Software Engineering, Jinling Institute of Technology, Nanjing, China; 4 Institute of Agricultural Resources and Environment, Jiangsu Academy of Agricultural Sciences, Nanjing, China

**Keywords:** biochar, rice-wheat rotation system, SOC, water-fertilizer interaction, yield

## Abstract

Rational management of coupled carbon (C), nitrogen (N), and water (W) inputs (C−N−W) is essential for the sustainability of rice−wheat rotation systems. Using a soil column pot experiment, we investigated the interactive effects of C−N−W on soil organic carbon (SOC) and crop grain yields. Two straw-derived wet biochars—wheat straw biochar (Cw) and rice straw biochar (Cr)—were applied only during the rice season. Three nitrogen levels were tested for both crops: N0 (no N), N1 (20% reduced N with a 3:7 ratio of quick-release to slow-release urea), and N2 (conventional N with a 4:6 ratio of quick-release to slow-release urea). Two irrigation regimes were applied: W1 (shallow flooding for rice/conventional irrigation for wheat) and W2 (alternate wetting-drying for rice/reduced irrigation for wheat). The control (CK) received no N and no biochar under W2 irrigation. All treatments significantly increased SOC in the whole system. C−N−W management enhanced SOC by 15.4%–23.9% during the rice season and by 1.6%–26.8% at wheat harvest. The optimal rotational rice/wheat SOC was achieved under Cr-W2N1 (8.03/8.11 g/kg) and Cr-W1N1 (7.86/8.22 g/kg). Wheat-season SOC was positively correlated with wheat root and shoot dry weights (*p* < 0.05). Compared with CK, certain C-N-W treatments increased rice grain yield by 1.3% to 136.3% and wheat grain yield by 6.2% to 14.6%. The highest rice yield (12.31×10^3^ kg/ha) was obtained under Cw-W1N2, and the highest wheat yield (2.80×10^3^ kg/ha) under Cr-W1N1. Random Forest analysis identified nitrogen management and stem biomass as variables closely associated with rice yield (*p* < 0.01), with Cw biochar being favorable; wheat grain yield depended on water−mediated growth modulated by preceding−crop management. Correlation analysis showed that rice dry biomass and effective panicle number were significantly correlated with wheat dry biomass and effective panicle number (*p* < 0.05). Comprehensive evaluation showed that Cr-W1N1 was the most favorable treatment, increasing rice–wheat yields and soil organic carbon and generating positive annual effects, thus offering an effective pathway for sustainable intensification through integrated C−N−W optimization.

## 1 Introduction

The rice–wheat rotation system serves as a cornerstone agricultural model for global food security (Kumari et al., 2011; Mohidem et al., 2022; Han et al., 2023). Under appropriate field management (e.g., rational fertilization and suitable irrigation), the rice–wheat rotation system can sustain high productivity while enhancing soil organic matter and soil organic carbon (SOC) (Zhu, 2024; Zhou et al., 2024). Effective nitrogen (N) management is paramount for the rice–wheat rotation system (Li et al., 2023). Achieving high productivity in the rice–wheat rotation system relies critically on the synergistic optimization of key resources, including carbon (C), N, and water (W) (Kumari et al., 2011; Mohidem et al., 2022; Han et al., 2023). These elements directly shape the growth and yield of the current-season crop. Moreover, through their interactions and soil legacy effects—whereby previous crop seasons influence soil properties and subsequently affect following crops—they profoundly impact soil health and long-term productivity of the rice–wheat rotation system (He, 2024). However, intensive agriculture can reduce soil organic matter, degrade soil structure, and decrease soil fertility (Glaser et al., 2002). Improper fertilization practices may impair soil quality, exacerbate environmental pollution, and harm human health (Li et al., 2023). Therefore, refined resource management is essential for the sustainable intensification of rice–wheat rotation systems.

Water management plays a key role in regulating the soil environment and crop growth in the rice–wheat rotation system. Soil moisture significantly influences the decomposition of organic matter (Moyano et al., 2012). Notably, soil moisture strongly regulates soil organic matter (SOM) decomposition, making ecosystem C fluxes highly sensitive to soil water conditions (Moyano et al., 2012; Yang et al., 2015). Consequently, different irrigation regimes have varying effects on root development and nutrient uptake in rice and wheat. Compared with traditional continuous flooding, the alternate wetting and drying irrigation (AWDI) strategy cyclically improves soil aeration, promotes rice root growth, and stimulates soil microbial activity, thereby mobilizing nutrients and enhancing plant nutrient uptake (Zhang et al., 2014; Wang Q. K. et al., 2017; Lin et al., 2021; Fa et al., 2024). By modifying the root zone environment, AWDI directly affects the current-season crop—for example, by promoting aboveground and belowground biomass accumulation in rice (Lin et al., 2021)—and influences soil organic matter dynamics through accelerated rhizosphere turnover and repeated wetting-drying cycles (Dijkstra and Cheng, 2007; Cheng, 2009). However, the effects of water management are context-dependent. Their impacts on crop growth and yield vary with soil type, climatic conditions, and cultivar, leading to outcomes that range from yield increases to decreases (Tabbal et al., 2002; Won et al., 2005; Yao et al., 2012). Importantly, irrigation not only affects the current growing season but also generates previous-crop effects on subsequent seasons. For example, irrigation patterns in the rice growing season can significantly alter soil microbial communities, and these changes persist into the fallow period, influencing greenhouse gas emissions (Martínez-Eixarch et al., 2022; Echeverría-Progulakis et al., 2025). They can also exert short-term legacy effects on soil bacterial communities at the start of the following wheat season (Tian et al., 2022). Furthermore, long-term paddy-upland rotation alters soil structure—a key physical legacy change that affects water infiltration and nutrient movement during the dry season (Liang et al., 2023).

Nitrogen management is crucial for ensuring high yield and quality of rice and wheat, as certain changes in soil N availability—such as low N input or high N input that alters the C/N ratio of organic materials and suppresses microbial activity—can inhibit organic matter decomposition (Wang et al., 2015; Chen et al., 2012; Hassett et al., 2009; Entwistle et al., 2013). Inhibiting SOC mineralization increases the content of various organic carbon components in soil aggregates, thereby suppressing soil respiration and promoting soil carbon sequestration (Wang T. T. et al., 2017; Zhong et al., 2017). Optimizing nitrogen management strategies—such as using controlled-release N fertilizers or their rational combination with quick-acting N fertilizers—aims to better synchronize nutrient supply with crop demand, thereby improving nitrogen use efficiency (Cai and Guo, 2018; Zou et al., 2022). Moreover, rational N management effectively increases rice yield and quality within the rice–wheat rotation system (Ma et al., 2021). Nitrogen management also exhibits legacy effects from previous crop growth: the N rate and residual N forms from the preceding crop directly affect soil N supply in the subsequent season. This helps regulate N uptake and yield formation in the following rice or wheat crop (He, 2024; Yang et al., 2025). In the rice–wheat rotation system, utilizing residual N from the previous season can increase subsequent yield, whereas excessive N input in the previous season may have negative effects; therefore, optimizing the N application rate across the full rotation cycle is crucial (Liu et al., 2025). This legacy effect of previous crop growth not only influences nutrient supply but may also drive final yield by affecting key developmental traits—for example, during the grain number determination period—rather than solely through biomass accumulation over the entire growing period (Slafer et al., 2023).

Straw return and biochar are key exogenous carbon inputs. Although straw return directly adds C and nutrients, its effectiveness highly depends on synergy with W and N management during decomposition. If mismanaged, straw return can temporarily hinder growth due to nutrient competition between microorganisms and crops (Jin et al., 2020; Zhang et al., 2025). In contrast, straw-derived biochar—a highly stable C material—more persistently increases the SOC pool and creates a more favorable root-zone environment by improving soil structure and enhancing water and nutrient retention (Karhu et al., 2011; He et al., 2017). Exogenous C input profoundly influences the soil C cycle. As the substrate for soil respiration, SOM mineralization is regulated by substrate quantity and quality, which directly affects SOC composition, microbial and faunal communities, and ultimately regulates the carbon cycle (Kaštovská et al., 2010; Fang et al., 2019). Research has confirmed that biochar application alone increases paddy SOM content, and combining it with straw produces a synergistic effect (Xia et al., 2024). The soil C sequestration process involves SOM decomposition and transformation, the driving role of microorganisms, and physical protection by clay and aggregates (Zhang et al., 2020). A slight increase in SOC can significantly reduce atmospheric CO_2_ concentrations (Zhu et al., 2024). Changes in SOC content are the net result of the decomposition of the original C pool and the addition of new exogenous carbon (Xia et al., 2020). Furthermore, applying biochar during the rice season rather than the wheat season more effectively improves nitrogen use efficiency and yield of the subsequent wheat crop and more strongly inhibits ammonia volatilization (Zhang et al., 2022). Long-term experiments have revealed complex effects. For example, biochar’s inhibitory effect on ammonia volatilization is lasting, but its regulation of nitrous oxide emissions shows seasonal divergence: it is effective during the rice season but differs during the wheat season. This highlights the need to comprehensively evaluate the agronomic and environmental effects of biochar across the full rotation cycle (He et al., 2022). However, the specific mechanisms by which aged biochar from the rice season affects subsequent wheat growth, yield, and SOM status still require in-depth investigation (Chen et al., 2023).

Previous studies have investigated the effects of water–nitrogen (W-N) and carbon–nitrogen (C-N) interactions on rice soil and plant growth (Xu et al., 2020; Chen et al., 2022). Rational fertilization and water management significantly reduce SOM decomposition, increase organic matter accumulation, and lower carbon gas emissions (Yan and Xia, 2015; Atere et al., 2020). The interaction between controlled-release N fertilizer and long-term straw return improves wheat yield and reduces N loss by regulating the soil microbial community and organic nitrogen composition (Zhou et al., 2025). Meanwhile, these influences are often transmitted between seasons via the “legacy effects of previous crop growth” (He, 2024; Jin et al., 2025; Shi and Ye, 2024). The synergy among C, N, and W is crucial for rice and wheat production and soil C sequestration (Rodriguez et al., 2019). However, understanding the coupled regulatory mechanisms of C (biochar), N (type and rate), and W (irrigation mode) on the productivity of the rice–wheat rotation system is still lacking. In particular, it remains unclear how interaction management strategies between C, N, and W (C-N-W) synergistically determine SOC content and yield components at harvest through the superposition of current- and previous-season effects. Indeed, individual management of C, N, or W can profoundly affect SOC and yield of both current and subsequent crops by altering the soil’s physicochemical and biological properties in the rice–wheat rotation system. In this study, a factorial experiment was established to evaluate the effects of different levels of biochar (C), nitrogen fertilizer types and rates (N), and irrigation regimes (W), representing a coupled C-N-W management strategy, on the rice–wheat rotation system. Based on this, this study proposed two core hypotheses: (1) In the rice–wheat rotation system, the optimized C-N-W coupling management strategy (combining biochar addition, appropriate N fertilization type and rate, and a suitable irrigation regime) improves SOC content and grain yields, along with promoting good plant growth at harvest in both seasons. (2) Previous rice management significantly influences subsequent wheat soil SOC and plant growth in the subsequent wheat season. To test these hypotheses, this study systematically investigates the effects of different C-N-W (carbon, nitrogen, and water) management combinations across the full rotation cycle on SOC, crop growth indicators (plant height, root dry weight, and stem and leaf dry weight), and final yield (effective panicle number and grain yield) at harvest for both rice and wheat. The goal is to identify reasonable C–N–W coupling management strategies that enhance SOC and yield in the rice–wheat rotation system.

## 2 Materials and methods

### 2.1 The tested soil and two straw-derived wet biochars

The tested soil (0–60 cm) was collected from a rice–wheat rotation system (E 118°5′, N 32°0′) and was mixed, ground, and passed through a 10-mesh sieve before being used for the soil column experiment. The basic properties of the tested soil were as follows: pH 8.32, soil organic carbon (SOC) 6.48 g/kg, alkali-hydrolyzable nitrogen (AN) 65.27 mg/kg, available phosphorus (AP) 36.09 mg/kg, and available potassium (AK) 179.0 mg/kg.

Two wet biochars derived from rice straw and wheat straw were prepared according to the method described by Chen et al. (2025). First, rice straw segments (3–5 cm) and wheat straw segments (5–8 cm, moisture <10%) were wrapped in aluminum foil and pyrolyzed in a lithium feldspar crucible (wall thickness ≥1 cm) at 400 °C ± 10°C (crucible wall temperature 320 °C–360 °C) for 5 min. The wall temperature was then reduced to 280 °C–290 °C for 10 min before heating was stopped. After 15 min, the foil package was transferred to an iron pan floating on water and rapidly cooled with ice packs for 10–15 min. The resulting biochar was sealed in a bag. The biochar was aged for 24 h, then mixed with diluted oxidant A (84 disinfectant with effective Cl 3.5%–5.0% w/v:purified water = 1:5 v/v) at a ratio of 500 g biochar to 5 L oxidant for 24 h of slow oxidation. Subsequently, 100 mL of fresh lemon juice was added, and the mixture was stored for another 24 h. The slurry was irradiated with electromagnetic waves for 1 h (stirred every 20 min; layer thickness ≤20 cm), followed by microwave heating for 3 min and germicidal light-wave treatment for 1 min. After cooling for 3.5 h, the material was filtered through gauze. The filter residue was sealed and frozen at –20 °C ± 5 °C for 12 h, then thawed to obtain the final wet biochar, which was stored at 2 °C–4 °C. The prepared wet biochars from rice straw and wheat straw exhibited total C contents of 459.0 and 449.3 g/kg, total N contents of 16.5 and 10.9 g/kg, pH values of 7.8 and 8.6, particle diameters of 0.25 and 0.45 cm, specific surface area increases of 10.6% and 15.5%, and moisture contents of 75.5% and 84.5%, respectively.

### 2.2 Experimental design

**Figure 1:**
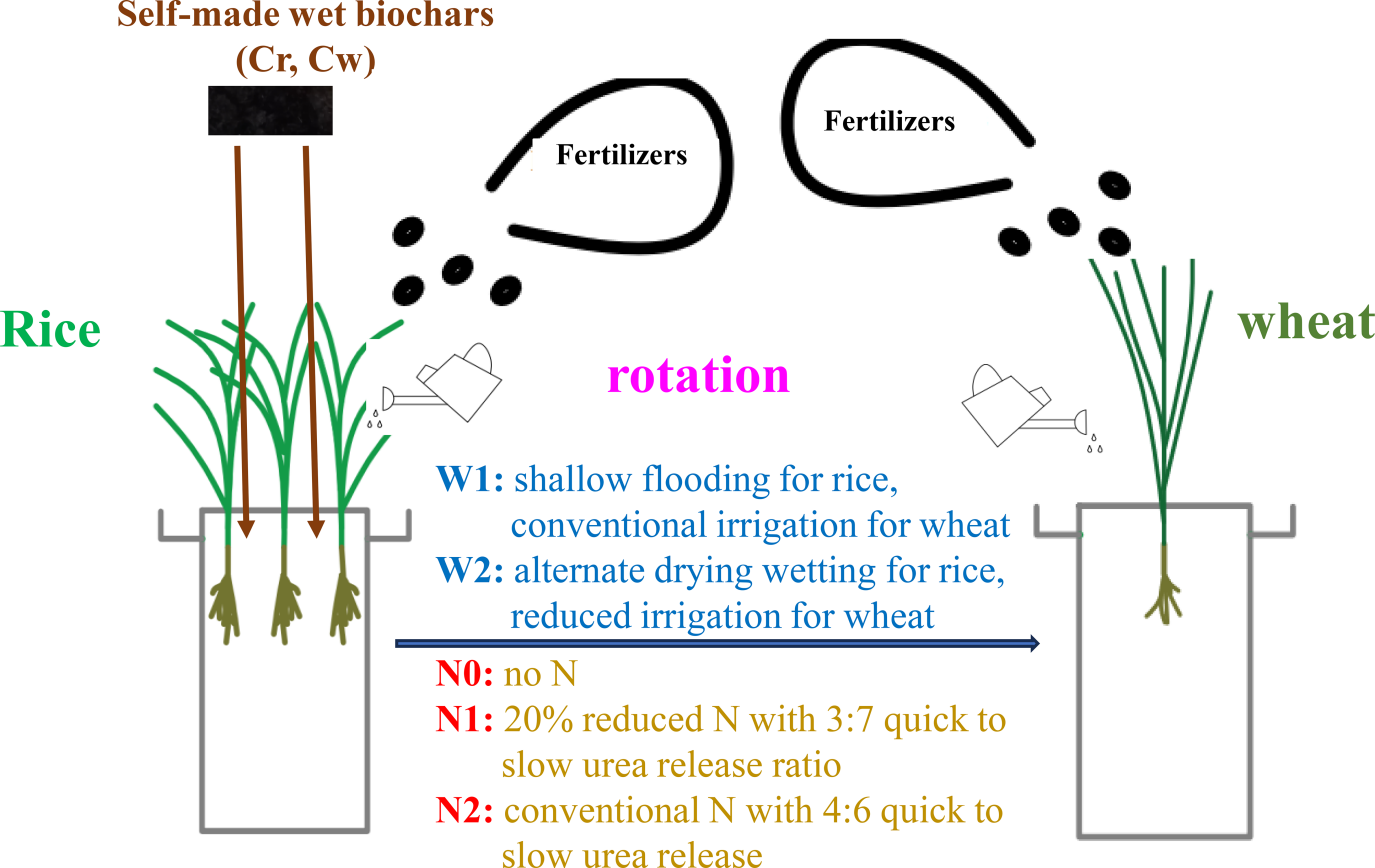
The conceptual diagram of the soil column pot experimental design.

A soil column pot experiment was initiated on 4 July 2024, with the rice cultivar “Nanjing 46”. Cylindrical pots (diameter: 0.3 m; height: 1.0 m) were filled with a base layer 70–75 cm deep using the tested soil. The base layer weighed approximately 50 kg per pot (air-dry weight basis) and contained no added biochar or fertilizer. After the base layer was added, the soil was watered to saturation and allowed to settle overnight. A plow layer (15–20 cm deep) was then formed by mixing 12.5 kg of the same air-dried, sieved paddy soil with the respective biochar and fertilizer treatments according to the experimental design (**Figure 1**). This mixture was uniformly placed on top of the base layer. Rice seedlings were transplanted into each pot (three holes per pot, three uniform seedlings per hole, seedling height 12.0–15.0 cm). After transplanting, all pots were watered to field capacity and then maintained according to the different irrigation regimes. Each column received one of three N management treatments (**Figure 1**), namely, N0 (0 kg N/ha), N1 (240.0 kg N/ha, 80% of conventional), and N2 (300.0 kg N/ha, conventional), combined with two irrigation systems and the two wet biochars. N1 and N2 consisted of quick-acting urea nitrogen (QAF-N) and slow-release urea nitrogen (SRF-N) at ratios of 3:7 and 4:6, respectively. QAF-N refers to conventional urea that releases nitrogen rapidly after application, whereas SRF-N releases nitrogen gradually over an extended period. The two were blended at specific ratios (3:7 for N1 and 4:6 for N2) to synchronize nitrogen availability with crop demand. Three controlled-release urea formulations with release periods of 60 days (45% N), 90 days (44% N), 120 days (43% N) were blended in specific proportions of total nitrogen. Under treatment N1, the proportions were 30%, 30%, and 10%; under treatment N2, the proportions were 20%, 20%, and 20%, respectively. Basal diammonium phosphate (18% N, 46% P_2_O_5_) was applied at 145.6 kg/ha and KCl (60% K_2_O) at 111.7 kg/ha. These were applied uniformly and mixed with the soil and biochar before rice transplanting. Two water management regimes were applied during the rice season (**Figure 1**). W-r1 maintained shallow water depths of 1 cm, 1 cm, and 0.5 cm from transplanting to reviving, tillering, and jointing-booting, respectively, then maintained moist soil conditions. W-r2 alternated wet-dry irrigation (AWDI) and maintained higher water depths of 3.0 cm, 3.0 cm, and 1.5–3.0 cm during the same stages. Mid-season drainage was initiated when tiller numbers reached 80% of the target, and cyclic wetting and drying was applied after flowering. All plots were drained 10 days before harvest. Rice straw-derived wet biochar (Cr) and wheat straw-derived wet biochar (Cw) were applied at 6.40 g/kg soil. The control (CK; C0-W2N0) received no biochar or nitrogen fertilizer and was managed under W2. All treatments had three replicates.

Rice was harvested on 23 November 2024. Subsequently, wheat (cultivar “Yangmai 25”) was direct-seeded into the same pots on 23 December 2024, with 18 holes per pot and three seeds per hole. The types and amounts of N, P, and K fertilizers applied to wheat grown in the same pots were identical to those applied to rice and were applied directly to the soil surface. However, no additional biochar was applied. After sowing, the soil was brought to field capacity. The soil surface was then covered with a layer (<0.3 cm thick) of 5.0–8.0 cm wheat straw segments. The surface was lightly sprinkled with water every 2–3 days to maintain surface moisture, and soil moisture was maintained at 60%–70% of field capacity for one month. The straw mulch was removed after 80% seedling emergence, with less than 5% of the straw residue remaining. Subsequent wheat irrigation followed the water management regimes applied to the preceding rice crop (**Figure 1**). Specifically, W-w1 (local standard practice) irrigated the pot treated by W-r1, whereas W-w2 (55%–80% of W-w1) irrigated the pot treated by W-r2. Under W-w1, soil moisture was maintained at approximately 80% of field water saturation capacity throughout the different growth stages. When soil moisture fell below this level, the irrigation amount was calculated using the FAO farmland water balance method. Under W-w2, soil moisture was maintained at 80% of that under W-w1. Accordingly, the actual irrigation amount under W-w2 ranged from 55% to 80% throughout the different growth stages. In practice, the total irrigation amount for wheat under the W-w2 treatment was approximately 73% of that under the W-w1 treatment. Before each irrigation event, soil moisture content was measured using a soil moisture meter (TR-8D, Beijing Shunkeda Technology Co., Ltd.). Thus, the composite water regimes were defined as W1 (W-r1 + W-w1) and W2 (W-r2 + W-w2) for the rice–wheat rotation system. Irrigation was withheld at the onset of maturity, approximately for 15–20 days before harvest. Wheat was subsequently harvested on 24 May 2025.

### 2.3 Indicator measurement

Soil samples were collected at crop harvest from the 0–20 cm soil layer using a stainless steel soil auger (5 cm inner diameter). From each experimental soil column, five cores were taken following a five−point sampling pattern and mixed to form a composite sample. After removal of visible plant residues and stones, the composite samples were air−dried, sieved (through a 2 mm sieve), and stored for subsequent analysis of soil organic carbon and pH. Soil pH was measured in a 1:2.5 soil-water suspension using a PHS-3C pH meter (Yu et al., 2020; Chen et al., 2022). SOC was determined according to the Chinese national standard method HJ 615-2011 (Ministry of Ecology and Environment of the People's Republic of China, 2011). Soil AN was quantified by alkaline hydrolysis diffusion with 1.0 M NaOH (Li et al., 2022). Soil AP and AK were determined using sodium bicarbonate−Mo−Sb anti-spectrophotometry and flame photometry, respectively, according to the Chinese national standard method HJ 704-2014 (Ministry of Ecology and Environment of the People's Republic of China, 2014).

Plant height (H) was measured with a ruler at physiological maturity. At harvest, the plants were carefully separated into roots, stems (including leaves), and grains. All separated components were then oven-dried to constant weight at 70 °C. The dry weights of roots (RDW) and stems (SDW) were recorded individually. Grain dry weight per pot was determined to calculate grain yield (Y). Total C and total N contents of the biochars were measured using a TOC analyzer (Multi N/C3100, Germany). Biochar pH, particle diameter, specific surface area increase, and moisture content were determined following the methods described in Yu et al. (2020).

### 2.4 Comprehensive evaluation index

Principal component analysis (PCA) was conducted on all indicators, including SOC, Y, H, RDW, and SDW for both rice and wheat, to determine their weights. The weight of each indicator was quantified as the sum of its factor loadings across the retained principal components. Principal components were selected based on eigenvalues greater than 1 and a cumulative variance contribution rate exceeding 80%. Subsequently, each indicator was normalized to a score ranging from 0 to 1 (Idowu et al., 2008; Yang et al., 2024). For indicators following the “more is better” principle, the scoring function was applied according to (**Equation 1**) (Yang et al., 2024).

(1) $$S=(V-V_{min})/(V_{max}-V_{min})$$

Where *S* is the score of a specific indicator, *V* is the measured value of the indicator, and *V_max_* and *V_min_* are the maximum and minimum values of the indicator among the treatments, respectively.

A comprehensive evaluation index (CI) was subsequently established for each treatment to assess the overall balance between SOC and plant growth across the entire rotation system. The CI was calculated as the weighted sum of the individual indicator scores according to (**Equation 2**).

(2) $$\text{CI}=\frac{\sum_{i=2}^{n}(s_{i}w_{i})}{\sum_{i=2}^{n}(w_{i})}$$

where *S_i_* is the score of the *i*th indicator, *w_i_* is the weight of the *i*th indicator, and *n* represents the number of indicators.

### 2.5 Data analysis

All data were initially processed using Microsoft Excel 2021 and then visualized with Origin 2024. All data are presented as means ± standard deviations (SD, n = 3). Statistical analysis was performed using IBM SPSS Statistics 27.0 (IBM Corp., Armonk, NY, USA). Treatment differences were assessed using one-way analysis of variance (ANOVA) followed by Duncan’s *post-hoc* test (*p* < 0.05). Pearson correlation analysis and two-way ANOVA were used to evaluate the individual effects of carbon (C), nitrogen (N), and water (W), together with their interactions (C × W, C × N, W × N, and C × W × N) (Chen et al., 2022). A random forest (RF) model was used to rank the importance of growth factors influencing rice yield (RY) and wheat yield (WY). Variables with a percent increase in mean squared error (%IncMSE) value greater than zero were retained as significant contributors to the model’s predictive accuracy (Feng et al., 2026).

## 3 Results

### 3.1 Effect of C-N-W treatments on soil organic carbon

Rice soil organic carbon contents at rice harvest showed no significant differences the C-N-W treatments (*p* > 0.05, **Table 1**). However, compared with the initial soil (6.48 g/kg), the SOC values under all treatments increased by a range of 15.4% (Cw-W2N1, 7.48 g/kg) to 23.9% (Cr-W2N1, 8.03 g/kg). On average, Cr treatments produced slightly higher mean SOC increases (20.9%) than Cw treatments, which showed an average increase of 18.7%. Compared with C0-W2N0 (7.83 g/kg), the following treatments showed higher rice SOC contents: Cw-W1N1, Cw-W2N0, Cr-W1N1, Cr-W2N0, and Cr-W2N2. N1 generally showed the most favorable effects, especially under Cr. Additionally, the W2 regime demonstrated greater increases in SOC than W1.

**Table 1:** Soil organic carbon conditions at rice and wheat harvest times.

Treatment	RSOC (g/kg)	CPr (%)	WSOC (g/kg)	CPw (%)	CR (%)
Cw-W1N0	7.63 ± 0.26a	17.7	7.39 ± 0.49abcd	13.9	–3.2
Cw-W1N1	7.93 ± 0.49a	22.4	6.92 ± 0.67de	6.6	–12.8
Cw-W1N2	7.61 ± 0.31a	17.3	6.97 ± 0.31de	7.6	–8.3
Cw-W2N0	8.02 ± 0.64a	23.6	6.59 ± 0.10e	1.6	–17.8
Cw-W2N1	7.48 ± 0.23a	15.4	7.57 ± 0.41abc	16.7	1.2
Cw-W2N2	7.51 ± 0.27a	15.8	7.35 ± 0.39bcd	13.4	–2.1
Cr-W1N0	7.71 ± 0.32a	18.8	6.89 ± 0.27de	6.3	–10.6
Cr-W1N1	7.86 ± 0.45a	21.1	8.22 ± 0.45a	26.8	4.7
Cr-W1N2	7.61 ± 0.46a	17.4	7.39 ± 0.49abcd	14.0	–2.9
Cr-W2N0	7.87 ± 0.27a	21.3	7.46 ± 0.59abc	15.1	–5.2
Cr-W2N1	8.03 ± 0.37a	23.9	8.11 ± 0.44ab	25.1	1.0
Cr-W2N2	7.96 ± 0.14a	22.8	7.91 ± 0.11ab	22.1	–0.6
C0-W2N0	7.83 ± 0.05a	20.7	7.53 ± 0.57abc	16.2	–3.8

Lowercase letters in the same column indicate significant differences among the different C-N-W treatments based on Duncan’s test (*p* < 0.05). Values are the means ± SD (n = 3). RSOC: rice soil organic carbon content. WSOC: soil organic carbon content. The remaining indicators were calculated as CPr = (RSOC – 6.48) × 100/6.48, CPw = (WSOC – 6.48) × 100/6.48, CR = (WSOC – RSOC) × 100/RSOC at the same plot. “–” indicates a reduced proportion. The following abbreviations are the same.

Wheat soil SOC contents at wheat harvest differed significantly among the C-N-W treatments (*p* < 0.05), with increases ranging from 1.6% (Cw-W2N0, 6.59 g/kg) to 26.8% (Cr-W1N1, 8.22 g/kg) relative to the initial soil. Similarly, Cr treatments produced slightly higher average wheat SOC increases (18.2%), whereas Cw treatments averaged only 10.0%. The following treatments showed greater wheat SOC increases than C0-W2N0 (7.53 g/kg): Cw-W2N1, Cr-W1N1, Cr-W2N1, and Cr-W2N2. Among these, Cw-W2N1, Cr-W1N1, and Cr-W2N1 showed net increases of 1.2%, 4.7%, and 1.0%, respectively, compared with the corresponding rice SOC contents. N1 consistently produced the greatest increases in wheat SOC, and the W2 regime also showed superior effects on wheat SOC.

### 3.2 Effect of C-N-W treatments on grain yield and effective panicle number

**Figure 2:**
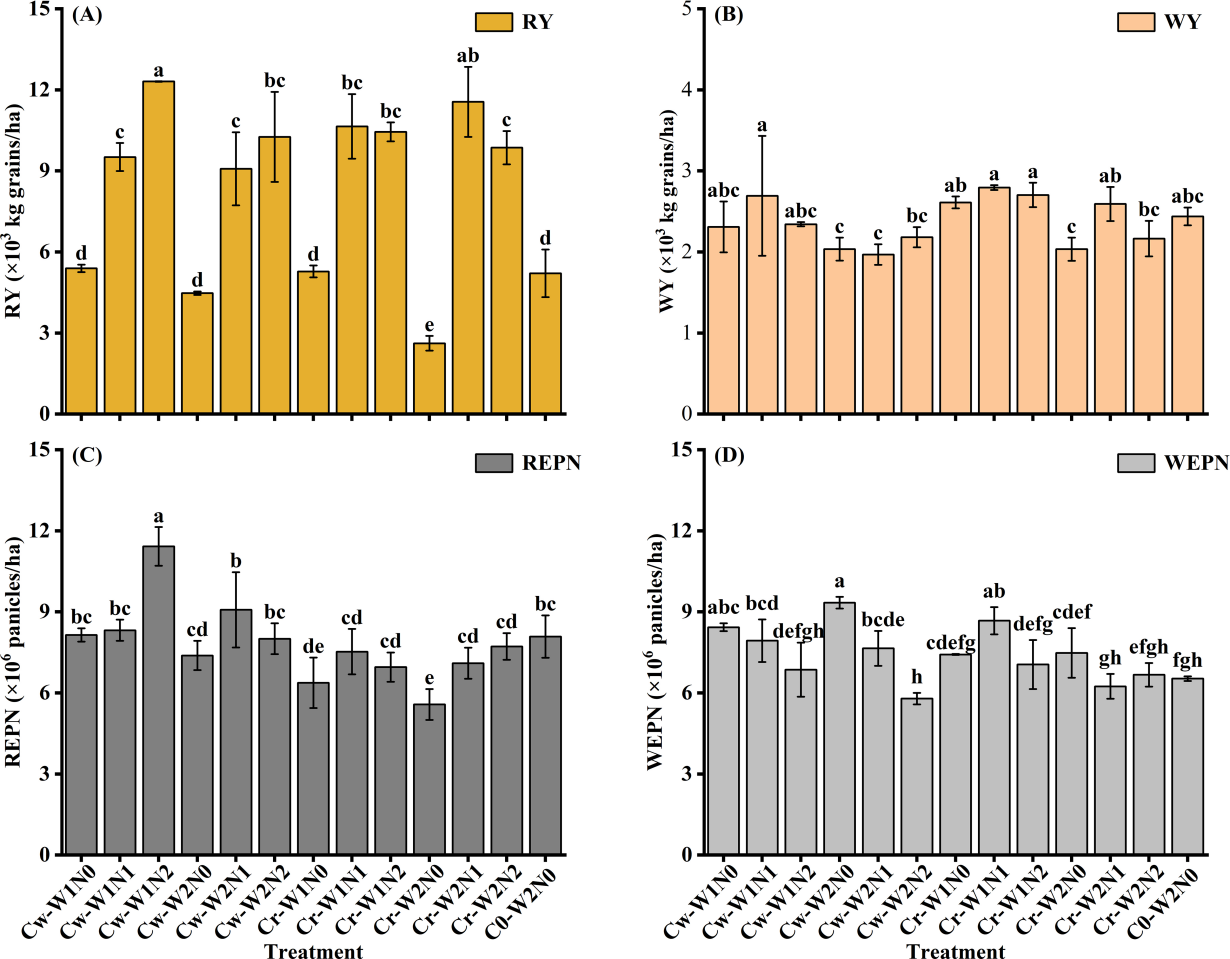
Conditions of grain yields and effective panicle numbers in rice–wheat rotation under C-N-W coupled treatments. **(A)** rice grain yield (RY); **(B)** wheat grain yield (WY); **(C)** rice effective panicle number (REPN); and **(D)** wheat effective panicle number (WEPN). Lowercase letters in the same column indicate significant differences among the different C-NW treatments based on Duncan’s test (*p* < 0.05). Values are the means ± SD (n = 3). The following abbreviations are the same.

Rice grain yield and wheat grain yield both showed significant differences across the C-N-W treatments (*p* < 0.05, **Figures 2A, B**). Only Cw-W2N0 and Cr-W2N0 exhibited significantly lower rice grain yield values than C0-W2N0 (*p* < 0.05, **Figure 2A**). Cw-W1N2 and Cr-W2N0 achieved the maximum and minimum rice grain yields, respectively, at 12.31 × 10^3^ kg/ha and 2.62 × 10^3^ kg/ha (*p* < 0.05). On average, Cw treatments produced slightly higher mean rice grain yield (8.50 × 10^3^ kg/ha) than Cr treatments (8.40 × 10^3^ kg/ha). Additionally, N2 showed the most favorable effects on rice grain yield under Cw, whereas N1 demonstrated greater effects under Cr. The W1 regime exhibited more favorable effects on rice grain yield under both biochars. Similar positive effects of N1 and W1 were observed for wheat grain yield (**Figure 2B**). However, only Cw-W1N1 (2.69 × 10^3^ kg/ha), Cr-W1N0 (2.61 × 10^3^ kg/ha), Cr-W1N1 (2.80 × 10^3^ kg/ha), Cr-W1N2 (2.70 × 10^3^ kg/ha), and Cr-W2N1 (2.59 × 10^3^ kg/ha) treatments produced higher wheat grain yield values than C0-W2N0 (2.44 × 10^3^ kg/ha). Rice effective panicle number and wheat effective panicle number both showed significant differences among the C-N-W treatments (*p* < 0.05, **Figures 2C, D**). All Cw-W1 treatments and Cw-W2N1 exhibited higher rice effective panicle number values than C0-W2N0 (**Figure 2C**). Cw-W1N2 achieved the maximum value of 11.42 × 10^6^ panicles/ha. Cr-W2N0 obtained the minimum value of 5.57 × 10^6^ panicles/ha (*p* < 0.05). On average, Cw produced a higher mean rice effective panicle number (8.73 × 10^6^ panicles/ha) than Cr (6.87 × 10^6^ panicles/ha). N1 showed the most favorable effects on rice effective panicle number under Cw-W2 and Cr-W1, whereas N2 showed greater effects under Cw-W1 and Cr-W2. N0 produced the lowest values for rice effective panicle number across all biochar and irrigation regimes. However, wheat effective panicle number showed the opposite pattern, with N0 generally producing higher values (**Figure 2D**). Cw-W2N0 achieved the highest value at 9.34 × 10^6^ panicles/ha. Only Cw-W2N2 and Cr-W2N1 exhibited lower wheat effective panicle numbers than C0-W2N0 (6.53 × 10^6^ panicles/ha). Additionally, Cw produced a higher mean wheat effective panicle number (7.67 × 10^6^ panicles/ha) than Cr (7.26 × 10^6^ panicles/ha). Moreover, wheat effective panicle number under Cw and Cr-W2 generally decreased with increasing nitrogen application. Meanwhile, the W1 regime generally produced higher wheat effective panicle numbers than the W2 regime.

### 3.3 Effect of C-N-W treatments on root and stem dry weights

**Figure 3:**
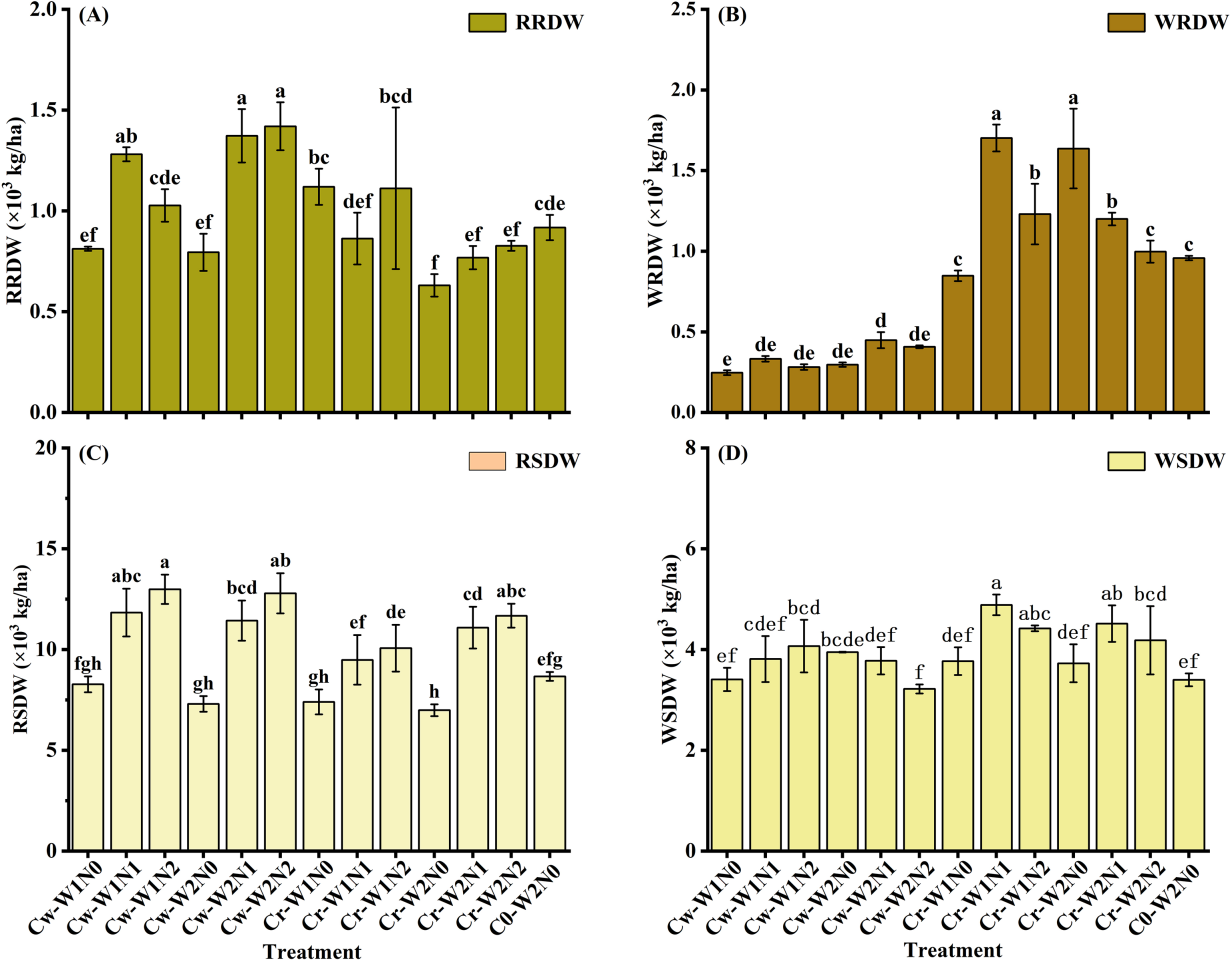
Conditions of dry matter in rice–wheat rotation under C-N-W coupled treatments. **(A)** rice root dry matter (RRDW); **(B)** wheat root dry matter (WRDW); **(C)** rice stem dry matter (RSDW); and **(D)** wheat stem dry matter (WSDW). The following abbreviations are the same.

Rice root dry weight and wheat root dry weight both varied significantly among the C-N-W treatments (*p* < 0.05, **Figure 3**). Cw-W2N2 and Cw-W2N1 achieved the highest rice root dry weights, at 1.42 × 10^3^ kg/ha and 1.37 × 10^3^ kg/ha, respectively (*p* < 0.05, **Figure 3A**). In contrast, Cr-W2N0 had the minimum rice root dry weight, at 0.63 × 10^3^ kg/ha. Furthermore, Cw-W1N1, Cw-W1N2, Cr-W1N0, and Cr-W1N2 also showed higher rice root dry weight values than C0-W2N0 (0.92 × 10^3^ kg/ha). On average, Cw produced a higher mean rice root dry weight (1.12 × 10^3^ kg/ha) than Cr (0.89 × 10^3^ kg/ha). Nitrogen fertilizers generally increased rice root dry weight under Cw and Cr-W1, whereas N0 and N2 showed similarly positive effects. Additionally, the W2 regime significantly increased rice root dry weight under Cw, whereas the W1 regime exhibited better effects under Cr. All W2 treatments under Cr produced the lowest rice root dry weights. For wheat, Cr-W1N1 (1.70 × 10^3^ kg/ha) and Cr-W2N0 (1.64 × 10^3^ kg/ha) produced the maximum wheat root dry weight (*p* < 0.05, **Figure 3B**), whereas Cw-W1N0 produced the minimum (0.25×10^3^ kg/ha). All Cr treatments exhibited higher wheat root dry weights than those under Cw. N1 showed the most favorable effects, producing the highest wheat root dry weight values under most treatments except under Cr-W2. The W2 regime showed higher average wheat root dry weight values than the W1 regime under each biochar treatment.

Rice shoot dry weight and wheat shoot dry weight both differed significantly among the C-N-W treatments (*p* < 0.05, **Figures 3C, D**). Cw-W1N2 and Cw-W2N2 achieved the highest rice shoot dry weights, at 12.99 × 10^3^ kg/ha and 12.79 × 10^3^ kg/ha, respectively (*p* < 0.05, **Figure 3C**). However, Cr-W2N0 had the minimum value, at 6.99 × 10^3^ kg/ha (*p* < 0.05). Furthermore, only Cw-W1N0, Cw-W2N0, Cr-W1N0, and Cr-W2N0 produced lower rice shoot dry weights than C0-W2N0 (8.67 × 10^3^ kg/ha). On average, Cw produced a higher mean rice shoot dry weight (10.77 × 10^3^ kg/ha) than Cr (9.45 × 10^3^ kg/ha). Nitrogen application significantly increased rice shoot dry weight, with N2 being the most effective treatment under each C-W combination. The W1 regime exhibited more favorable effects on rice shoot dry weight under Cw, whereas the W2 regime showed better effects under Cr. Cr-W1N1 produced the highest wheat shoot dry weight, at 4.89 × 10^3^ kg/ha (*p* < 0.05, **Figure 3D**). Meanwhile, Cw-W2N2 resulted in the lowest value, which was lower than that of C0-W2N0 (3.40 × 10^3^ kg/ha). Cr biochar consistently produced a higher mean wheat shoot dry weight (4.25 × 10^3^ kg/ha) than Cw (3.71 × 10^3^ kg/ha). N1 was the most effective treatment under Cr for improving wheat shoot dry weight. However, nitrogen application increased wheat shoot dry weight under Cw-W1, whereas it decreased wheat shoot dry weight under Cw-W2. The W1 regime was superior for both Cw and Cr in terms of average values.

### 3.4 Effect of C-N-W treatments on plant height

**Figure 4:**
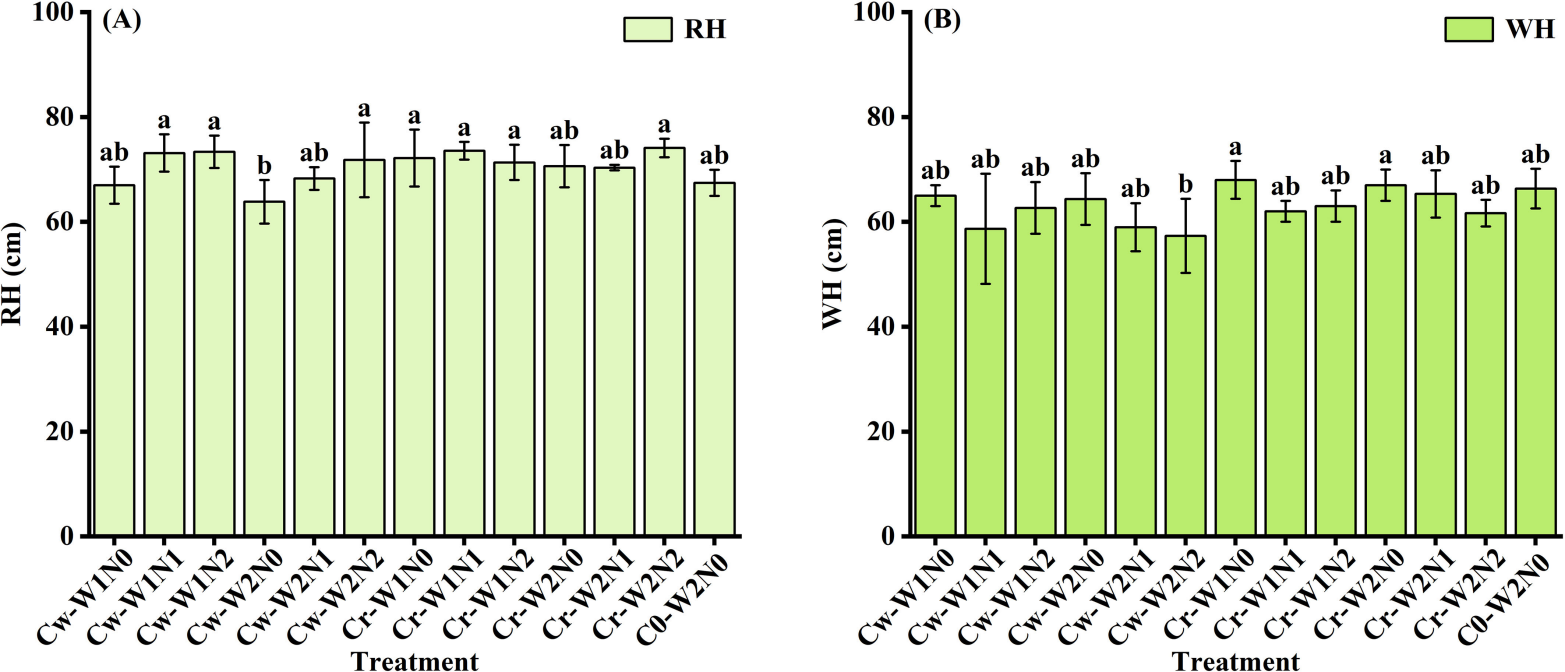
Conditions of plant height of rice (**A**: RH) and wheat (**B**: WH) at harvest. The following abbreviations are the same.

Rice plant height and wheat plant height at harvest both varied significantly across all C-N-W treatments (*p* < 0.05, **Figures 4A, B**). Cr-W2N2 showed the highest rice plant height, at 74.11 cm (*p* < 0.05, **Figure 4A**). Conversely, Cw-W2N0 showed the lowest rice plant height, at 63.80 cm, which was lower than C0-W2N0 (67.44 cm). On average, Cr generally resulted in taller plants (average of 72.03 cm) across most N-W combinations, except W1N2, compared with Cw (average of 69.57 cm). Rice plant height increased with increasing nitrogen application under Cw. However, under Cr, N2 and N1 produced the highest and lowest rice plant heights, respectively. The W1 regime tended to produce taller rice plants, with higher average values (71.16 cm under Cw and 72.36 cm under Cr) than W2 (67.97 cm under Cw and 71.70 cm under Cr) under each biochar treatment. For wheat, Cr-W1N0 and Cr-W2N0 showed the highest wheat plant heights, at 68.00 cm and 67.00 cm, respectively (*p* < 0.05, **Figure 4B**), both of which exceeded C0-W2N0 (66.33 cm). Conversely, Cw-W2N2 showed the lowest wheat plant height (57.30 cm). Similarly, Cr treatments generally resulted in taller plants, with a higher average wheat plant height (64.50 cm) than Cw (61.17 cm). Nitrogen application reduced wheat plant height compared with N0 under the same C-W treatment. However, the W1 regime under Cw enhanced wheat plant height, while the W2 regime showed more favorable effects under Cr.

### 3.5 Correlation analysis results

**Figure 5:**
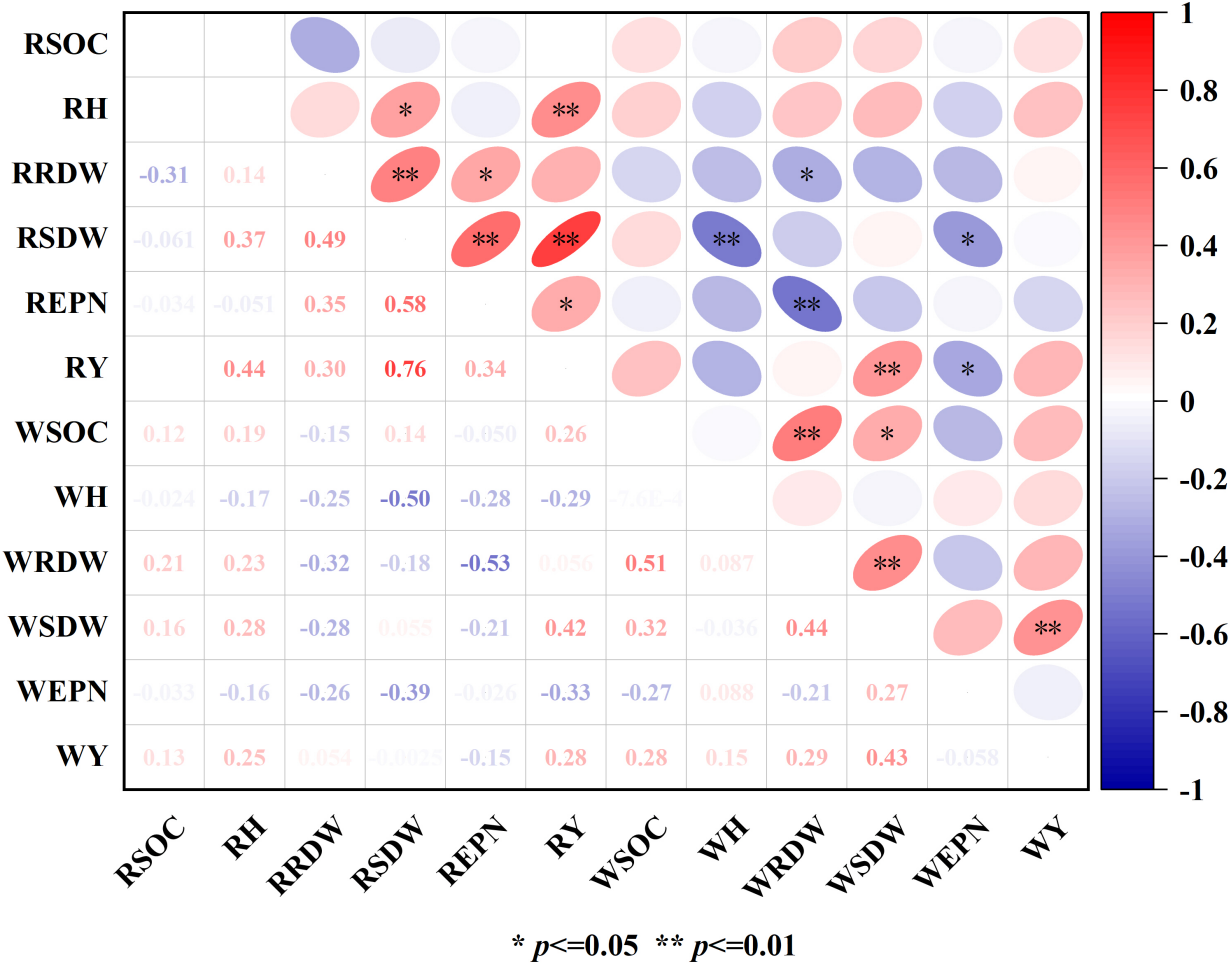
Results of Spearman correlation analysis between all indicators (n = 39). * and ** indicate Spearman correlation significance levels at 0.05 and 0.01, respectively. The red number with a shadow indicate a positive correlation. The blue number with a shadow indicate a negative correlation. The following abbreviations are the same.

Spearman correlation analysis revealed complex relationships between agronomic practices and crop growth in the rice–wheat rotation system (**Figure 5**). Rice soil organic carbon did not exhibit any significant relationships with rice plant growth indicators (*p* > 0.05). Rice plant height showed significant and highly significant relationships with rice shoot dry weight (r = 0.37, *p* < 0.05) and rice yield (r = 0.44, *p* < 0.01). Rice root dry weight also showed highly significant and significant relationships with rice shoot dry weight (r = 0.49, *p* < 0.01) and rice effective panicle number (r = 0.35, *p* < 0.05), respectively. It also showed a positive correlation with rice yield (r = 0.30). Moreover, rice shoot dry weight showed highly significant (*p* < 0.01) positive correlations with rice effective panicle number (r = 0.58) and rice yield (r = 0.76). Rice effective panicle number was also positively correlated with rice yield (r = 0.34, *p* < 0.05).

For wheat, wheat soil organic carbon showed significant positive correlations with wheat root dry weight (r = 0.51, *p* < 0.01) and wheat shoot dry weight (r = 0.32, *p* < 0.05). Wheat shoot dry weight was the primary factor associated with wheat yield (r = 0.43, *p* < 0.01), and wheat root dry weight also contributed positively (r = 0.29). Wheat root dry weight showed a strong positive relationship with wheat shoot dry weight (r = 0.44, *p* < 0.01). The correlation between wheat effective panicle number and wheat yield was not significant (r = −0.06, *p* > 0.05), whereas wheat plant height showed no significant relationships with other wheat plant growth indicators (*p* > 0.05).

Furthermore, rice growth and yield showed no direct relationships with wheat yield (*p* > 0.05). However, rice root dry weight (r = −0.32, *p* < 0.05) and rice effective panicle number (r = −0.53, *p* < 0.01) showed significant negative correlations with wheat root dry weight. Rice shoot dry weight also showed significant negative correlations with wheat plant height (r = −0.50, *p* < 0.01) and wheat effective panicle number (r = −0.39, *p* < 0.05). However, rice yield showed a highly significant positive relationship with wheat shoot dry weight (r = 0.42, *p* < 0.01), but a negative correlation with wheat effective panicle number (r = −0.33, *p* < 0.05). Rice yield also showed a positive correlation with wheat yield (r = 0.28).

### 3.6 Random forest model results

**Figure 6:**
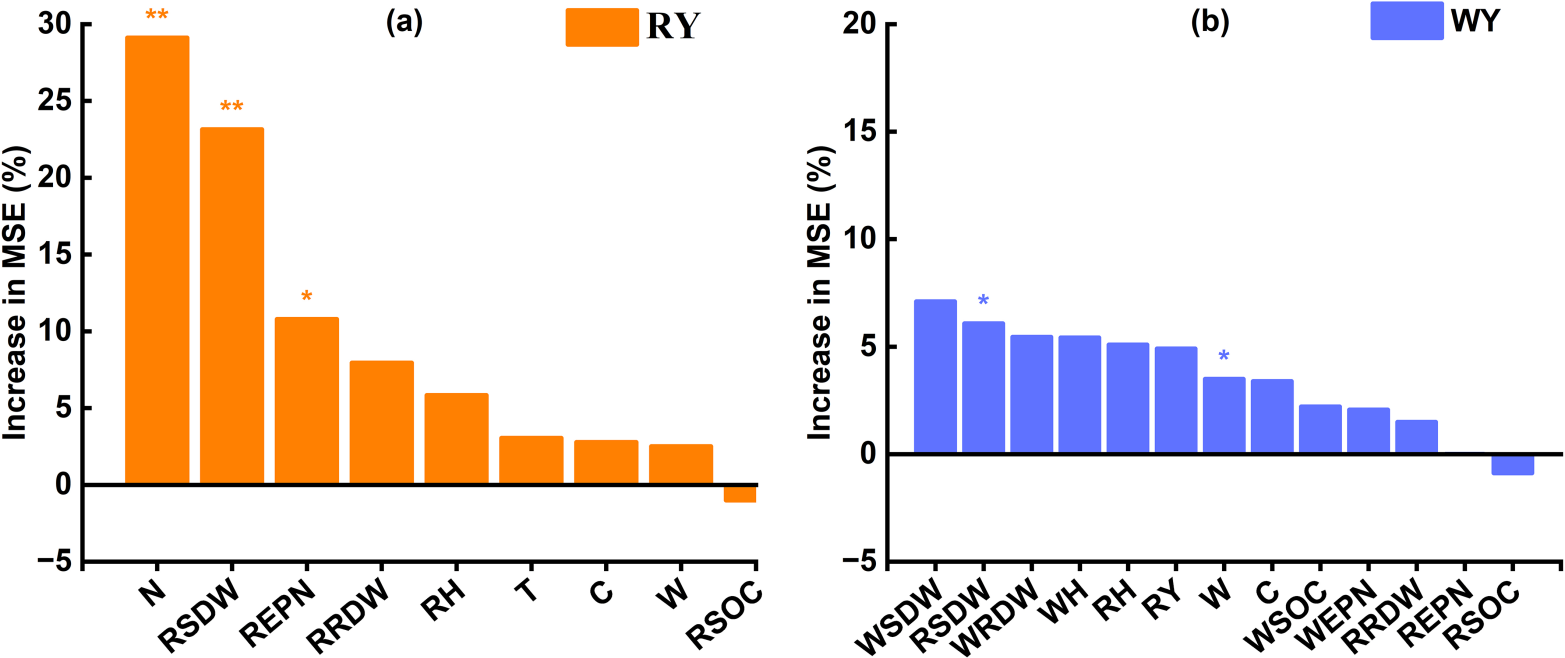
Results of the random forest model for RY **(a)** and WY **(b)**. * and ** indicate significance levels at 0.05 and 0.01, respectively. T: all C-N-W treatments; C: biochar type; N: nitrogen fertilization treatments, including N0, N1, and N2; andW: irrigation systems (two irrigation regimes). The following abbreviations are the same.

The relative importance of predictors for rice and wheat yield was assessed using Random Forest analysis (**Figure 6**). The dominant factors influencing rice yield were nitrogen treatments and rice shoot dry weight (*p* < 0.01, **Figure 6A**), in descending order, with increases in mean squared error (MSE) of 29.12% and 23.15%, respectively. Rice effective panicle number also significantly contributed to rice yield, with an increase in MSE of 10.8% (*p* < 0.05). In contrast, the key drivers of wheat yield were rice shoot dry weight and water (W) treatment (*p* < 0.05, **Figure 6B**), with increases in MSE of 6.08% and 3.50%, respectively. Other variables did not significantly contribute to either crop yield.

Rice root dry weight and rice plant height showed increases in MSE greater than 5.00% for rice yield. Biochar (C) and W were the most important shared drivers for both crops, with positive %IncMSE values. Moreover, wheat shoot dry weight had the highest %IncMSE value (7.10%) for wheat yield. Wheat root dry weight, wheat plant height, and rice plant height also showed increases in MSE greater than 5.00% for wheat yield. Only rice soil organic carbon exhibited negative %IncMSE values for both crop yields. Among the other soil indicators, soil pH was also measured after rice and wheat harvests. The results showed minimal variation across treatments (ranging from 8.12 to 8.43 after rice harvest and 8.02 to 8.45 after wheat harvest, compared with the initial pH of 8.32; **Supplementary Table 1**). Given the small magnitude of these differences and the highly buffered alkaline soil, pH was not considered a major explanatory factor for the observed treatment effects on crop yield.

## 4 Discussion

### 4.1 Response of soil organic carbon to C-N-W coupling treatments in the rice–wheat rotation system

In this study, all C-N-W coupling treatments increased soil organic carbon (SOC) by 15.4%–23.9% relative to the baseline value of 6.48 g/kg, with no significant differences among treatments (*p* > 0.05, **Table 1**). This rapid, treatment-insensitive increase is consistent with Karhu et al. (2011), who reported that large inputs of labile carbon from organic amendments dominated short-term SOC dynamics regardless of specific management details. The immediate SOC increase observed here is directly attributable to the application of wet biochars during the rice season.

Rice straw biochar (Cr) produced higher average rice-season SOC (7.9 g/kg) than wheat straw biochar (Cw; 7.72 g/kg). This feedstock-dependent difference aligns with Liu et al. (2021), who demonstrated that biochars with higher C/N ratios decompose more slowly, leading to greater SOC accumulation. The Cr biochar had a higher total C/N ratio (459.0/16.5) than Cw (449.3/10.9), which likely reduced microbial decomposition rates. Reduced nitrogen treatment (N1), especially under W2 irrigation (alternate wetting-drying for rice and reduced irrigation for wheat), showed superior effects on SOC. This finding is supported by Zhou et al. (2022, 2023), who reported that slow-release urea blends (3:7 quick-to-slow release ratio) promote microbial turnover and residue formation. Similarly, Gao et al. (2023) found that such N management enhances soil aggregation, whereas Lu et al. (2025) observed increases in active organic carbon and the carbon pool management index. Additionally, flooded anaerobic conditions during the rice season likely reduced SOC mineralization rates through effects on soil microbial biomass, as reported by Liu et al. (2021) and Hu et al. (2024).

Although biochar was not reapplied during the wheat season, significant treatment effects on wheat-season SOC emerged (*P* < 0.05). Wheat-season SOC increased by 1.6%–26.8% compared with the initial soil, consistent with cross-seasonal legacy effects reported by He (2024) and Zhang et al. (2022). Positive carryover effects were observed in CrW1N1 (+4.7% relative to rice SOC at harvest), CrW2N1 (+1.0%), and CwW2N1 (+1.2%). In contrast, the control (CK) showed a decline of 3.8%. These results indicate that rice-season biochar application enhances subsequent wheat-season SOC only when combined with appropriate N and water management, consistent with Martínez-Eixarch et al. (2022) and Tian et al. (2022). Statistical analysis revealed that although three-way (C × N × W) and two-way (W × C) interactions were not significant (*p* > 0.05), the individual factors C, N, and W significantly affected wheat-season SOC under C × N and W × N interactions (*p* < 0.05; **Supplementary Tables 2, 3**). This finding suggests that two-factor interactions, rather than higher-order interactions, drive the SOC responses observed across the two growing seasons.

### 4.2 Response of crop growth and grain yield to C-N-W coupling treatments in the rice–wheat rotation system

Significant variations across all growth parameters (*p* < 0.05, **Figures 2**-**4**) confirm that C-N-W practices and their interactions drive markedly different productivity responses in rice and wheat. These findings align with Chen et al. (2023), who demonstrated that rice–wheat rotation productivity depends on synergistic optimization of C, N, and W resources. For rice, Random Forest analysis identified N management as a driver factor with high relative importance for yield (%IncMSE = 29.12%, *p* < 0.01; **Figure 6A**). Rice shoot dry weight (RSDW; %IncMSE = 23.15%, *p* < 0.01) and effective panicle number (REPN; %IncMSE = 10.79%, *p* < 0.05) were also significant drivers. These results corroborate Cai and Guo (2018) and Ma et al. (2021), who established that optimized N management is fundamental to rice productivity. The main physiological pathway operates through enhanced aboveground biomass accumulation (RSDW–rice yield: r = 0.76, *p* < 0.01; **Figure 5**), which directly contributes to yield and promotes effective panicle formation (RSDW–REPN: r = 0.58, *p* < 0.01). Notably, SOC showed no significant correlation with rice growth indicators (*p* > 0.05, **Figure 5**), and the RF model indicated a negative %IncMSE value for rice SOC on rice yield (−1.01%, **Figure 6A**). This counterintuitive result is consistent with Chen et al. (2023), who reported that short−term rapid SOC increases (e.g., from fresh biochar−induced microbial activity) may temporarily immobilize nutrients or alter soil physical conditions without immediate yield benefits. In contrast, during the wheat season, wheat SOC was positively correlated with wheat root dry weight (WRDW; r = 0.51, *p* < 0.01) and wheat shoot dry weight (WSDW; r = 0.32, *p* < 0.05; **Figure 5**), indicating that improved soil carbon from prior biochar application supported wheat root and shoot development.

Biochar effects were feedstock-dependent with distinct seasonal patterns. In the rice season, wheat-straw biochar (Cw) generally induced superior growth compared to rice-straw biochar (Cr), particularly under W1 (shallow flooding for rice and conventional irrigation for wheat). The highest rice yield (12.31 × 10^3^ kg/ha) and RSDW (12.99 × 10^3^ kg/ha) were both achieved under Cw-W1N2 (**Figures 2A**, **3C**). In addition, Cw promoted higher average values than Cr for rice yield (8.50 vs. 8.40 × 10^3^ kg/ha), REPN (8.73 vs. 6.87 × 10^6^ panicles/ha), rice root dry weight (1.12 vs. 0.89 × 10^3^ kg/ha), and RSDW (10.77 vs. 9.45 × 10^3^ kg/ha) (**Figures 2A, C**, **3A, C**). However, an opposite pattern emerged in the subsequent wheat crop. Cr applied in the rice season consistently promoted stronger wheat root development. Cr treatments produced the highest wheat root dry weights, with Cr-W1N1 reaching 1.70 × 10^3^ kg/ha and Cr-W2N0 reaching 1.64 × 10^3^ kg/ha, substantially outperforming Cw treatments and the control (**Figure 3B**). This enhanced root system under Cr likely contributed to the generally higher wheat yields in Cr treatments, with Cr-W1N1 achieving the maximum wheat shoot dry weight (4.89 × 10^3^ kg/ha; **Figure 3D**) and wheat yield (2.80 × 10^3^ kg/ha; **Figure 2B**). These feedstock-specific carryover effects have been previously reported by Zhang et al. (2022) and He (2024). RF analysis further confirmed that biochar management was a significant shared driver with positive importance for both crop yields (2.79% and 3.39% IncMSE, **Figure 6**). Irrigation effects were highly context-dependent. For rice under adequate N (N2), continuous shallow flooding (W1) generally supported higher yields than alternate wetting and drying (W2) (e.g., Cw-W1N2: 12.31 vs. Cw-W2N2: 10.34 × 10^3^ kg/ha; **Figure 2A**). This finding aligns with Zhang et al. (2014), who reported that stable water and nutrient availability under continuous flooding minimizes N losses. The stable water environment likely promotes sustained release of slow-release N (4:6 quick-to-slow urea release in N2), leading to better crop growth. However, under Cr biochar and reduced N (N1), the W2 regime produced outstanding rice yield (Cr-W2N1: 11.55 × 10^3^ kg/ha), significantly higher than its W1 counterpart (Cr-W1N1: 10.61 × 10^3^ kg/ha; **Figure 2A**). This suggests that alternate wetting and drying irrigation, known to improve soil aeration and stimulate root activity (Lin et al., 2021), may synergize with Cr properties to enhance N recovery under reduced N input. The W2 regime showed higher average wheat root dry weight values than W1 under each biochar (**Figure 3B**), and RF analysis identified water management as a key driver for wheat yield (%IncMSE = 3.50%, *p* < 0.05; **Figure 6B**).

Reduced N rate (N1, 20% less than conventional) under biochar amendments often produced yields comparable to or exceeding conventional N (N2) without biochar. For example, Cr-W2N1 achieved significantly higher rice yield than the C0-W2N0 control (**Figure 2A**). This superior effect of N1, particularly under W2, is consistent with Zhou et al. (2022, 2023), Gao et al. (2023), and Lu et al. (2025), who attributed improved yields to the priming effect of a slow-release urea blend (3:7 quick-to-slow release ratio) promoting microbial turnover and residues, soil aggregation, and active organic carbon.

### 4.3 Impacts of rice growth on succeeding wheat performance

Correlation analysis and RF models revealed significant impacts of rice growth on subsequent wheat performance. Rice yield showed a positive but non-significant correlation with subsequent wheat yield (r = 0.28, *p* > 0.05; **Figure 5**), and RF analysis identified rice shoot dry weight as a key driver of wheat yield (%IncMSE = 6.08%, *p* < 0.05; **Figure 6B**), suggesting that vigorous rice growth may create favorable conditions for the following wheat crop. Notably, certain rice-season treatments, particularly those involving rice-straw biochar (Cr), consistently enhanced wheat root development (e.g., CrW1N1: 1.70 × 10^3^ kg/ha; **Figure 3B**) and wheat yield (CrW1N1: 2.80 × 10^3^ kg/ha; **Figure 2B**). These benefits likely arise from improved soil physical structure and residual nutrient availability following rice cultivation, rather than from direct carryover of biochar per se.

Conversely, significant negative correlations were also observed (rice root dry weight vs. wheat root dry weight: r = −0.32, *p* < 0.05; rice root dry weight vs. wheat height: r = −0.50, *p* < 0.01; rice shoot dry weight vs. wheat effective panicle number: r = −0.39, *p* < 0.05). These negative relationships suggest that under certain conditions, a highly productive rice system may deplete soil resources or create residual allelopathic effects that limit subsequent wheat growth, consistent with cross-seasonal microbial and competitive effects reported by Tian et al. (2022).

Overall, the benefits of rice-season management on wheat productivity are treatment-specific. Among all treatments, CrW1N1 (rice-straw biochar + 20% reduced N with a 3:7 quick-to-slow urea release ratio + shallow flooding for rice/conventional irrigation for wheat) demonstrates the most consistent positive contribution from rice to wheat. This treatment not only achieves the second highest rice yield but also produced the highest wheat yield and wheat-season SOC (**Figure 2**, **Tables 1**, **2**). Furthermore, the CI results (**Table 2**) clearly show that while Cw-W1N2 excels in rice yield alone, its poor wheat−season performance (especially wheat SOC) makes it suboptimal for the whole−rotation system. Cr-W1N1, with the highest CI at 0.798, achieves the best balance across all indicators, particularly the critical cross−seasonal parameters of wheat yield and wheat SOC, while maintaining competitive rice yield. Therefore, considering soil organic carbon, grain yield, and auxiliary growth indicators collectively, CrW1N1 is identified as the optimal treatment for the integrated rice–wheat rotation system.

**Table 2:** The comprehensive evaluation index (CI) of each treatment.

Treatment	CI	Rank			
		RY	WY	RSOC	WSOC
Cr-W1N1	0.798	2	1	5	1
Cr-W2N1	0.765	1	4	1	2
Cr-W1N2	0.712	3	3	10	6
Cr-W2N2	0.689	5	8	3	4
Cw-W1N2	0.647	1	10	10	11
Cw-W1N1	0.612	6	2	2	13
Cw-W2N2	0.592	4	9	12	8
Cr-W1N0	0.561	11	5	8	12
Cw-W2N1	0.543	8	12	13	5
Cr-W2N0	0.528	13	10	4	3
C0-W2N0	0.511	9	7	6	7
Cw-W1N0	0.498	7	6	9	10
Cw-W2N0	0.465	12	11	7	9

RY, rice yield; WY, wheat yield; RSOC, rice SOC; WSOC, wheat SOC.

### 4.4 Limitations

This experiment included only one complete rice–wheat rotation cycle. Therefore, the long-term stability of the observed effects under different treatments remains unknown. Multi-year, multi-cycle experiments are needed to confirm whether the identified optimal treatment (Cr-W1N1) can sustain its positive effects on soil organic carbon and crop yields over time. In addition to SOC, other soil physicochemical properties, such as pH, were measured but showed minimal treatment variation (**Supplementary Table 1**). However, other properties, including total nitrogen, available phosphorus, bulk density, and microbial biomass, were not measured in this study, which limits our ability to fully explain the mechanistic pathways driving the observed crop responses. Future studies should incorporate a broader set of soil parameters to better understand the integrated effects of C−N−W management.

## 5 Conclusions

This study used a soil column pot experiment to demonstrate that the interaction of exogenous biochars (Cw and Cr), nitrogen regimes (N0, N1, and N2), and irrigation systems (W1 and W2)—collectively referred to as C-N-W management—drives rice–wheat productivity and soil organic carbon at harvest. Rice and wheat grain yields increased by 1.3% to 136.3% and 6.2% to 14.6%, respectively, compared with the control. The highest rice grain yield (12.31 × 10^3^ kg/ha) was observed under the Cw-W1N2 treatment, driven primarily by nitrogen management and stem biomass (%IncMSE = 29.12% and 23.15%, respectively; *p* < 0.01). The highest wheat grain yield (2.80 × 10^3^ kg/ha) was observed under the Cr-W1N1 treatment, which was associated with enhanced root growth promoted by legacy effects of previous crop growth. Cw (wheat-straw wet biochar) favored rice production, whereas Cr (rice-straw wet biochar) exerted legacy effects on subsequent wheat growth. However, negative correlations (e.g., rice root dry weight vs. wheat root dry weight: r = –0.32) suggested that rice root growth under C-N-W management during the rice season may limit growth in the subsequent wheat season. C−N−W management significantly increased SOC across the entire system by 15.4%–23.9% at rice harvest and by 1.6%–26.8% at wheat harvest. The optimal SOC enhancements were observed under Cr-W2N1 (rice SOC: 8.03 g/kg; wheat SOC: 8.11 g/kg) and Cr-W1N1 (rice SOC: 7.86 g/kg; wheat SOC: 8.22 g/kg). Wheat-season SOC was positively correlated with wheat root dry weight (*p* < 0.01) and wheat shoot dry weight (*p* < 0.05). In summary, treatment Cr-W1N1 in this study can serve as the optimal treatment for improving soil organic carbon and crop yield in rice–wheat rotation system. However, ongoing and follow-up experiments are being conducted to evaluate the multi-year carryover effects and long-term sustainability of the recommended C−N−W management strategy.

## Data Availability

The original contributions presented in the study are included in the article/**Supplementary Material**, further inquiries can be directed to the corresponding author/s.
