# Supplementary material for: Interactive effects of biochar, nitrogen fertilizer, and irrigation on SOC and crop yield in a rice-wheat rotation system
Source: Front Plant Sci. 2026 Jun 30;17:1859708. doi: 10.3389/fpls.2026.1859708 (PMC13366217; doi:10.3389/fpls.2026.1859708)
Supplement: Supplementary file 1 [file Table1.docx]

**Supplementary material**

**Exploration of Biochar, N fertilizers and irrigation interacted effects on Soil Organic Carbon Contents and Crop Grain Yields in Rice-Wheat Rotation System**

**Authors**:

Danyan Chen ^1^, Yuanyuan Feng ^2^, Hailing Li ^1^, Ya Liu ^3^, Hongbo Ma^4^, Xianju Xu^4^, Xiangyu Kong ^1^, Heng Qi ^1^, Yuxuan Zhang ^1^, Jiaying Wang ^1^, Jingze Ma ^2^, Zhenyue Li ^1^, Jiameng Qian^1^, Wei Zhou ^4, *^

*^1^ College of Horticulture, Jinling Institute of Technology, Nanjing 210038, China*

*^2^ Co-Innovation Center for Sustainable Forestry in Southern China, College of Forestry, Nanjing Forestry University, Nanjing 210037, China*

*^3^ School of Software Engineering, Jinling Institute of Technology, Nanjing 211169, China*

*^4^ Institute of Agricultural Resources and Environment, Jiangsu Academy of Agricultural Sciences, Nanjing, 210014, China*

**^*^Corresponding Author:**

Wei Zhou, No. 50 Zhonglingjie Street, Jiangsu Academy of Agricultural Sciences, Nanjing 210014, Jiangsu, China; zwei@jaas.ac.cn (W.Z.)

**Table S1** Soil pH values at rice and wheat harvest times.

| Treatment | pH | |
| --- | --- | --- |
|  | Rice soil | Wheat soil |
| Cw-W1N0 | 8.31±0.09ab | 8.02±0.35bc |
| Cw-W1N1 | 8.36±0.15a | 8.15±0.16bc |
| Cw-W1N2 | 8.31±0.09ab | 8.08±0.06c |
| Cw-W2N0 | 8.37±0.09a | 8.12±0.02bc |
| Cw-W2N1 | 8.39±0.09a | 8.18±0.04bc |
| Cw-W2N2 | 8.28±0.04abc | 8.16±0.08bc |
| Cr-W1N0 | 8.33±0.09a | 8.45±0.15a |
| Cr-W1N1 | 8.34±0.07a | 8.30±0.06ab |
| Cr-W1N2 | 8.12±0.08d | 8.29±0.02ab |
| Cr-W2N0 | 8.43±0.09a | 8.40±0.06a |
| Cr-W2N1 | 8.17±0.04bcd | 8.37±0.04a |
| Cr-W2N2 | 8.15±0.08cd | 8.44±0.11a |
| CK | 8.13±0.05cd | 8.30±0.05ab |

**Table S2.** Two factor interaction results of all investigated indexes under C and N managements.

| **Factors** | **C** | | **N** | | **C×N** | |
| --- | --- | --- | --- | --- | --- | --- |
|  | **rice** | **wheat** | **rice** | **wheat** | **rice** | **wheat** |
| Y | - | - | *** | - | ** | - |
| NS | *** | ** | ** | *** | - | * |
| SDW | *** | *** | *** | ** | - | * |
| RDW | ** | *** | ** | - | ** | - |
| H | - | - | * | * | - | - |
| SOC | - | ** | - | * | - | - |

C, N and C×N means effects of biochar or fertilizer or biochar-N fertilizer interaction by two-way ANOVA analysis, with ***, ** and *displays extremely significant at *p* < 0.001, 0.01 and 0.05, respectively. The ns displays no significant difference. The following abbreviations are the same.

**Table S3.** Two factor interaction results of all investigated indexes under W and N managements.

| **Factors** | **W** | | **N** | | **W×N** | |
| --- | --- | --- | --- | --- | --- | --- |
|  | **rice** | **wheat** | **rice** | **wheat** | **rice** | **wheat** |
| **Y** | - | * | *** | - | ** | - |
| **NS** | *** | - | * | *** | - | ** |
| **SDW** | *** | *** | *** | ** | - | * |
| **RDW** | *** | *** | ** | - | ** | - |
| **H** | - | * | ** | * | - | - |
| **SOC** | - | ** | - | * | - | - |

W, N and W×N means effects of watering or fertilizer or watering-N fertilizer interaction by two-way ANOVA analysis.
